# Characterization of an M-Cluster-Substituted Nitrogenase VFe Protein

**DOI:** 10.1128/mBio.00310-18

**Published:** 2018-03-13

**Authors:** Johannes G. Rebelein, Chi Chung Lee, Megan Newcomb, Yilin Hu, Markus W. Ribbe

**Affiliations:** a Department of Molecular Biology and Biochemistry, University of California, Irvine, California, USA; b Department of Chemistry, University of Basel, Basel, Switzerland; c Department of Chemistry, University of California, Irvine, California, USA; University of Massachusetts Amherst

**Keywords:** carbon monoxide, cofactor, hydrocarbons, molybdenum, nitrogenase, vanadium

## Abstract

The Mo- and V-nitrogenases are two homologous members of the nitrogenase family that are distinguished mainly by the presence of different heterometals (Mo or V) at their respective cofactor sites (M- or V-cluster). However, the V-nitrogenase is ~600-fold more active than its Mo counterpart in reducing CO to hydrocarbons at ambient conditions. Here, we expressed an M-cluster-containing, hybrid V-nitrogenase in *Azotobacter vinelandii* and compared it to its native, V-cluster-containing counterpart in order to assess the impact of protein scaffold and cofactor species on the differential reactivities of Mo- and V-nitrogenases toward CO. Housed in the VFe protein component of V-nitrogenase, the M-cluster displayed electron paramagnetic resonance (EPR) features similar to those of the V-cluster and demonstrated an ~100-fold increase in hydrocarbon formation activity from CO reduction, suggesting a significant impact of protein environment on the overall CO-reducing activity of nitrogenase. On the other hand, the M-cluster was still ~6-fold less active than the V-cluster in the same protein scaffold, and it retained its inability to form detectable amounts of methane from CO reduction, illustrating a fine-tuning effect of the cofactor properties on this nitrogenase-catalyzed reaction. Together, these results provided important insights into the two major determinants for the enzymatic activity of CO reduction while establishing a useful framework for further elucidation of the essential catalytic elements for the CO reactivity of nitrogenase.

## IMPORTANCE

This is the first report on the *in vivo* generation and *in vitro* characterization of an M-cluster-containing V-nitrogenase hybrid. The “normalization” of the protein scaffold to that of the V-nitrogenase permits a direct comparison between the cofactor species of the Mo- and V-nitrogenases (M- and V-clusters) in CO reduction, whereas the discrepancy between the protein scaffolds of the Mo- and V-nitrogenases (MoFe and VFe proteins) housing the same cofactor (M-cluster) allows for an effective assessment of the impact of the protein environment on the CO reactivity of nitrogenase. The results of this study provide a first look into the “weighted” contributions of protein environment and cofactor properties to the overall activity of CO reduction; more importantly, they establish a useful platform for further investigation of the structural elements attributing to the CO-reducing activity of nitrogenase.

## INTRODUCTION

Nitrogenase is an important metalloenzyme that catalyzes certain remarkable chemical transformations under ambient conditions (1). Catalysis by nitrogenase is enabled by ATP-dependent transfer of electrons from a reductase component to a catalytic component of the enzyme, followed by the subsequent reduction of substrates at the cofactor site of the catalytic component upon accumulation of sufficient electrons (2, 3). Using this two-component mechanism, the nitrogenase is capable of reducing nitrogen (N_2_) to ammonia (NH_3_), as well as carbon monoxide (CO) to hydrocarbons (e.g., propane [C_3_H_8_] and butane [C_4_H_10_]) (4, 5) at ambient conditions. Interestingly, these two reactions parallel the industrial Haber-Bosch and Fischer-Tropsch processes, respectively, which are used for large-scale production of ammonia and carbon fuels. However, in contrast to the energy-demanding industrial processes, the enzymatic reactions occur under ambient temperatures and pressures (6, 7). The unique features of the nitrogenase-catalyzed reactions make them fascinating subjects of study from a perspective of chemical energy while suggesting the potential of using these systems as prototypes for future development of biomimetic catalysts for energy- and cost-efficient production of useful chemical compounds.

The molybdenum (Mo)- and vanadium (V)-dependent nitrogenases are two homologous members of the nitrogenase family (8). Mainly distinguished by the presence of a different heterometal (i.e., Mo or V) at the cofactor site, the two nitrogenases comprise a pair of homologous component proteins: a homodimeric reductase component (*nifH*- or *vnfH*-encoded Fe protein), which contains a subunit-bridging [Fe_4_S_4_] cluster and an MgATP-binding site within each subunit; and a multimeric catalytic component (*nifDK*-encoded MoFe protein or *vnfDGK*-encoded VFe protein), which contains an 8Fe P-cluster species (P- or P*-cluster) at each α/β-subunit interface and a 7Fe/1Mo or 7Fe/1V cofactor species (M- or V-cluster) within each α subunit (9). Intriguingly, while biochemical, spectroscopic, and structural analyses reveal a striking resemblance between the Mo- and V-nitrogenases in terms of protein structure and cluster species (9, 10), the two nitrogenase systems are clearly distinct in their catalytic behaviors. Most notably, the Mo- and V-nitrogenases display significantly different reactivities toward the substrate CO, with the former showing a marginal activity of ~0.02 nmol of reduced carbon/nmol of protein/min, and the latter demonstrating a significantly increased activity at ~16 nmol of reduced carbon/nmol of protein/min—substantially higher than its Mo counterpart (4, 5). The observation of highly differential CO-reducing activities of two homologous nitrogenases has prompted us to define key features of these systems that contribute to this discrepancy in activity; in particular, the question of whether the protein environment or the cofactor species determines the reactivity of nitrogenase toward CO needs to be addressed, as knowledge in this regard represents the first step toward understanding the CO-reducing activity of nitrogenase for the potential applications of this reactivity in the future.

## RESULTS

**FIG 1:**
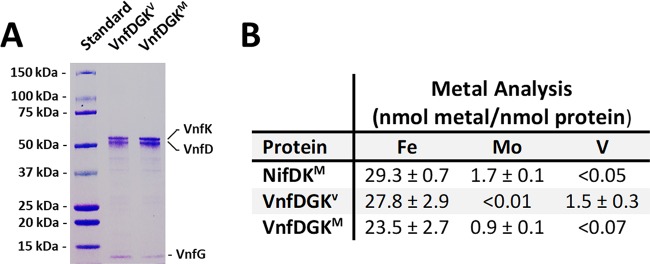
Subunit and metal compositions of VnfDGK^V^ and VnfDGK^M^. (A) SDS-PAGE analysis of VnfDGK^V^ and VnfDGK^M^. The molecular masses (in kilodaltons) of the protein standards are shown to the left of the gel. (B) Metal contents of NifDK^M^, VnfDGK^V^, and VnfDGK^M^.

To tackle the question in hand, the M- and V-clusters must be placed in the same protein environment for direct comparison. Using a genetically altered *Azotobacter vinelandii* strain, a pair of VFe proteins containing either the V- or M-cluster can be generated *in vivo* for this line of investigation. This *A. vinelandii* strain expresses a His-tagged form of VFe protein in a genetic background that contains deletions of (i) the *nifDK* genes, which encode the MoFe protein, and (ii) the *mod* genes, which encode the Mo uptake system (locus tag Avin_50650-Avin_50730 of the *A. vinelandii* DJ strain) (11–14). Using this *A. vinelandii* strain, a V-cluster-containing native form of the VFe protein (designated VnfDGK^V^) was produced *in vivo* when V was supplemented in the growth medium (Fig. 1A), where deletion of the Mo transporter prevented incorporation of trace Mo into the cofactor (12–14), whereas an M-cluster-containing hybrid form of the VFe protein (designated VnfDGK^M^) was produced *in vivo* when Mo was added in excess to the growth medium (Fig. 1A), where the uptake of Mo was accomplished by other transporter systems, such as those involving siderophores (15, 16).

**FIG 2:**
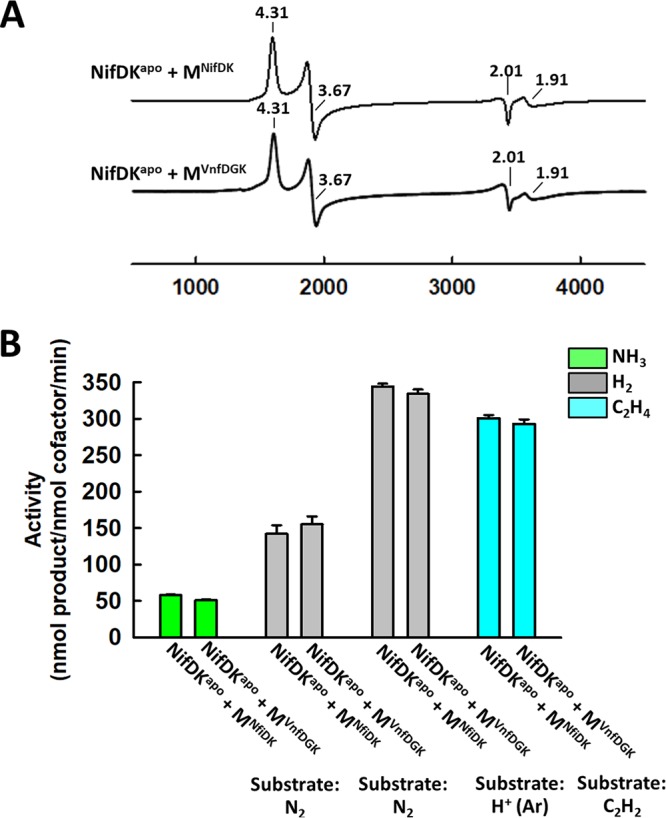
Spectroscopic and catalytic properties of the M-cluster extracted from VnfDGK^M^. (A and B) EPR spectra (A) and activity profiles (B) of the cofactor-deficient NifDK^apo^ protein reconstituted with the M-cluster extracted from NifDK (NifDK^apo^ + M^NifDK^) or VnfDGK^M^ (NifDK^apo^ + M^VnfDGK^). The *g* values are indicated in panel A. Activities are expressed as nanomoles of product per nanomole of cofactor per min in panel B.

Like the native VnfDGK^V^ protein, the VnfDGK^M^ hybrid consists of α, β, and δ subunits, although the δ subunit is present in a much reduced quantity in VnfDGK^M^ than in VnfDGK^V^ (Fig. 1A). Metal analysis reveals a metal content of 0.9 nmol Mo and less than 0.07 nmol V per nmol protein (Fig. 1B), suggesting that VnfDGK^M^ houses an Mo-containing cofactor in place of a V-containing species. Not too surprisingly, the heterologous incorporation of the Mo-containing cofactor into the VnfDGK scaffold is less efficient than the homologous incorporation of the V-containing cofactor, as the Mo content of VnfDGK^M^ supports the assignment of one M-cluster per protein, which is lower than the assignment of ~1.5 V-clusters per protein in the case of VnfDGK^V^. The identity of the cofactor species in VnfDGKM is confirmed by extracting the cofactor from VnfDGK^M^ into an organic solvent, *N*-methylformamide (NMF), and subsequently inserting it into the cofactor-deficient apo-NifDK (designated NifDK^apo^). As shown in Fig. 2A, the apo-NifDK protein reconstituted with the cofactor extracted from VnfDGK^M^ (designated M^VnfDGK^) exhibits EPR features (*g* = 4.31, 3.67, 2.01, and 1.91) identical to those of the NifDK^apo^ protein reconstituted with the cofactor extracted from the wild-type NifDK (designated M^NifDK^). Moreover, when combined with the reductase component (designated NifH), the M^VnfDGK^- and M^NifDK^-reconstituted NifDK^apo^ proteins demonstrate nearly indistinguishable substrate-reducing activities when N_2_, proton (H^+^) or acetylene (C_2_H_2_) is supplied as the substrate (Fig. 2B). Together, these observations establish VnfDGK^M^ as an M-cluster-containing counterpart of VnfDGK^V^.

**FIG 3:**
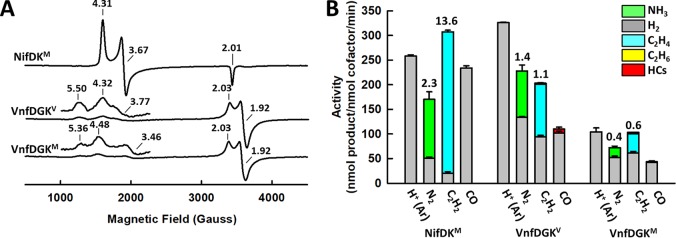
Spectroscopic and catalytic properties of NifDK^M^, VnfDGK^V^, and VnfDGK^M^. (A and B) EPR spectra (A) and activity profiles (B) of NifDK^M^, VnfDGK^V^, and VnfDGK^M^. Note the presence of the same *S* = 1/2 signal in the spectra of VnfDGK^V^ and VnfDGK^M^ (see panel A), which was assigned to the P*-cluster (i.e., a pair of [Fe_4_S_4_]-like clusters) in the case of VnfDGK^V^ (11). The *g* values are indicated in panel A, and the products are color coded in panel B. The substrates are indicated at the bottom of the bar chart, and the ratios of N_2_/H_2_ and C_2_H_4_/H_2_ generated in the reactions of N_2_ and C_2_H_2_ reduction are indictedabove the respective bars in panel B. HCs, hydrocarbons.

**FIG 4:**
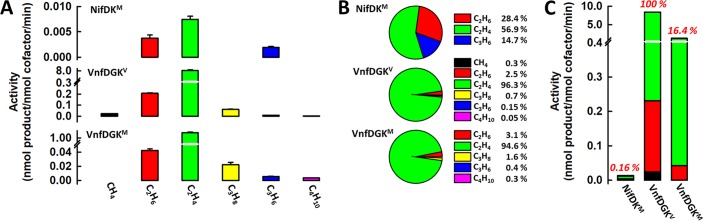
CO-reducing activities and product profiles of NifDK^M^, VnfDGK^V^, and VnfDGK^M^. (A to C) Individual activities of hydrocarbon formation (A), distributions of hydrocarbon products (B), and total activities of hydrocarbon product formation (C) by NifDK^M^, VnfDGK^V^, and VnfDGK^M^ when CO is supplied as a substrate. Activities are expressed as nanomoles of product per nanomole of cofactor per minute in panels A and C. The percentage activities of proteins are shown in red in panel C, with the total activity of VnfDGK^V^ set at 100% and those of NifDK^M^ and VnfDGK^M^ calculated accordingly. Note that there is a contribution of V to the activity of VnfDGK^M^ due to the presence of <0.07 nmol of V per nmol of VnfDGK^M^. However, the percentage contribution of V to the overall activity of VnfDGK^M^ cannot be conclusively determined due to the inaccuracy of V determination in this low concentration range.

Interestingly, the M-cluster in VnfDGK^M^ displays EPR features (*g* = 5.36, 4.48, and 3.46) similar to those of the native V-cluster in VnfDGK^V^ (*g* = 5.50, 4.32, and 3.77), both of which are clearly distinct from the EPR features of the native M-cluster in NifDK^M^ (*g* = 4.31 and 3.67) (Fig. 3A). This observation is interesting, as it highlights a strong impact of protein environment on the properties of the cofactor. Consistent with the observed similarity between their EPR features, VnfDGK^M^ seems to follow its native VnfDGK^V^ counterpart in terms of the overall product distribution patterns, demonstrating decreased NH_3_/H_2_ and C_2_H_4_/H_2_ ratios relative to those generated by NifDK^M^, the ability to generate C_2_H_6_ from C_2_H_2_ reduction that is absent from NifDK^M^, and higher activity than NifDK^M^ in producing hydrocarbons from CO reduction (Fig. 3B) (4, 5, 8, 11). The similarity between the CO reactivities of VnfDGK^M^ and VnfDGK^V^ is particularly striking. Both VnfDGK^M^ and VnfDGK^V^ reduce CO to hydrocarbons of up to C_4_ in length, whereas in comparison, NifDK^M^ has a narrower product profile comprising up to C_3_ hydrocarbons (Fig. 4A). Moreover, the product distribution profiles of VnfDGK^M^ and VnfDGKV are remarkably similar, with C_2_H_4_/C_2_H_6_ comprising 96.3%/2.5% and 94.6%/3.1%, respectively, of the total amounts of hydrocarbons generated by these proteins, displaying a clear tendency toward formation of the unsaturated C_2_ product (C_2_H_4_); in contrast, NifDK^M^ generates C_2_H_4_/C_2_H_6_ at 56.9%/28.4% of the total amounts of hydrocarbons, showing a preference for formation of the saturated C_2_ product (C_2_H_6_) (Fig. 4B). The protein environment, therefore, appears to “normalize” the product profiles of the M- and V-clusters in CO reduction once they are inserted into the same protein scaffold, VnfDGK. Further, the fact that VnfDGK^M^ is considerably more active than NifDK^M^ (by ~100-fold) in CO reduction illustrates the higher efficiency of the VnfDGK scaffold in catalyzing this reaction (Fig. 4C).

There is, however, a clear contribution of the cofactor properties to the CO-reducing activity, as VnfDGK^V^ is ~6-fold more active than VnfDGK^M^ in hydrocarbon formation, which demonstrates that the V-cluster is better tuned toward CO reduction than the M-cluster (Fig. 4C). Moreover, despite the “normalization” of the protein environment, the ability of VnfDGK^V^ to form detectable amounts of CH_4_ is not observed in the case of VnfDGK^M^ under the same experimental conditions (Fig. 4B). Given the absence of CH_4_ from the product profile of NifDK^M^ (Fig. 4B), this trait seems to be carried over to VnfDGK^M^ by the M-cluster, further highlighting the characteristics of the unique properties of the M-cluster in the reaction of CO reduction. Taken together, these results suggest a combined effect of protein environment and cofactor properties on the reactivity of nitrogenase toward CO: the protein scaffold has a significant impact on the overall activity of CO reduction (Fig. 4C, VnfDGK^M^ versus NifDK^M^), whereas the cofactor species fine-tunes the product profile of CO reduction while exerting a moderate impact on the overall activity (Fig. 4C, VnfDGK^V^ versus VnfDGK^M^). It is interesting to note that a “weighted” contribution of protein environment and cofactor properties to the CO-reducing activity can be derived from these comparisons, with (i) the ~100-fold difference that arises from the difference in protein scaffold and (ii) the ~6-fold difference that arises from the difference in cofactor species contributing collectively to an ~600-fold difference between the CO-reducing activities of Mo- and V-nitrogenases.

## DISCUSSION

The impact of protein environment on the CO reactivity of nitrogenase is intimately associated with the immediate surroundings of the cofactor that could play a significant role in the interactions between the cofactor and the substrate CO. The cofactor “pocket” in the recently reported crystal structure of VnfDGK is slightly more polar than its counterpart in NifDK, which may influence the electrochemical properties of the cofactor (17). Moreover, the cofactor captured in the crystal structure of VnfDGK has a “belt” sulfur substituted by a carbonate moiety (17). A comparison between the cofactor-binding sites in the crystal structures of VnfDGK and NifDK reveals comparable hydrogen bonding networks around the homocitrate moieties of the two cofactors but markedly different hydrogen bonding at the position where carbonate is bound to the V-cluster, which could contribute to the differences in the catalytic activities of the two proteins (17). Other than the cofactor environment, the P-cluster species, which mediates electron transfer to the cofactor, could also impact the CO reactivity of nitrogenase. While the P-cluster in the crystal structure of VnfDGK is determined to have the same [Fe_8_S_7_] structure as its counterpart in NifDK, there are additional electron densities at the P-cluster site that suggest the possible existence of an additional P-cluster conformation(s) that is not populated or captured in the specific redox state of the VnfDGK crystal (17). This observation is in line with the X-ray absorption spectroscopy (XAS)/extended X-ray absorption fine structure (EXAFS)-derived structure of the P-cluster of a cofactor-deficient VnfDGK, which suggests that this cluster assumes the conformation of a [Fe_4_S_4_]-like cluster pair in the solution state. It is likely, therefore, that the P-cluster of VnfDGK is capable of undergoing different conformational changes than those of its counterpart in NifDK upon redox changes. In this context, it is interesting to note that the P-cluster of the reduced, resting-state VnfDGK exhibits analogous *S* = 1/2 and *S* = 5/2 electron paramagnetic resonance (EPR) signals to the one-electron-oxidized, P^1+^ state of the P-cluster of NifDK (8, 18), which has been implicated in substrate turnover. Such a difference in redox states, likely associated with the conformational differences between the two P-cluster species, could very well impact the ability of the respective proteins to transfer electrons to their cofactor sites and, consequently, the catalytic activities of these proteins.

The impact of cofactor properties on the CO reactivity of nitrogenase, on the other hand, could stem from the presence of different heterometals in the M- and V-clusters. Interestingly, differential abilities of synthetic V- and Mo-containing compounds to reductively couple two CO moieties into functionalized acetylene ligands have been observed previously (19), which suggests a higher capacity of V (a first-row transition metal) than Mo (a second-row transition metal) in this type of reactions. While this observation may be used to account for the differential reactivities of V- and M-clusters toward CO, it remains unclear whether the heterometal directly participates in substrate reduction or exerts an indirect effect on the electronic/catalytic properties of the cofactor. Apart from the differential heterometal compositions of the V- and M-clusters, the presence of a carbonate moiety at the belt region of the V-cluster—a feature that is absent from any M-cluster structure reported so far—may also impact the nitrogenase reactivity (17). The observed substitution of a belt sulfide of the V-cluster by carbonate is interesting, as carbonate is a potential carbon substrate of this cofactor. However, the sulfide displaced by carbonate in the structure of the V-cluster is different than the sulfide equivalent displaced by CO in the structure of the CO-bound M-cluster (20). Moreover, a catalysis-dependent migration of belt sulfide has been suggested recently for the M-cluster, which could very well enable displacement of carbonate by a sulfide during substrate reduction in the case of the V-cluster (21). This proposal is also consistent with our XAS/EXAFS-derived structure of the isolated V-cluster, where a sulfide is modeled in place of carbonate in the belt region of this cofactor (22). The unlikely scenario that carbonate, a very weak ligand, has survived the cluster extraction procedure, along with the observation that the isolated V-cluster can be used to reconstitute cofactor-deficient proteins, suggests that a carbonate-free conformation of the V-cluster is likely the competent form in substrate reduction (22). Clearly, the origin and catalytic relevance of the carbonate moiety needs to be clarified before mechanistic interpretations can be made based on this finding.

The *in vitro* formation of an M-cluster containing the VnfDGK hybrid and analysis of its N_2_-reducing activity was reported earlier (23). However, the *in vivo* generation of this hybrid, which permits a direct comparison of the activities of hydrocarbon formation by the M- and V-cluster-containing VnfDGK proteins generated under cell growth conditions, has not been accomplished prior to the current study. Other than facilitating a direct assessment of the contributions of protein scaffold and cofactor species to the CO-reducing activity of nitrogenase, our *in vivo* generation of a heterologous form of VnfDGK that contains an M-cluster at its cofactor-binding site also sheds light on the regulation of nitrogenase expression and the biosynthesis of the “alternative” nitrogenase. It is interesting to note that, despite the deletion of the *mod*-encoded Mo uptake system (11–14) in *A. vinelandii*, the cells still manage to acquire sufficient Mo from a growth medium supplemented with excess Mo for the synthesis of M-clusters. In contrast to earlier suggestions (24), the expression of *vnf* genes in a *nifDK* deletion background is not suppressed by the amount of Mo taken up by this mechanism. Moreover, unlike NifEN that is specific for M-cluster synthesis (25, 26), VnfEN is apparently capable of synthesizing both M- and V-clusters for VnfDGK, further facilitating the formation of VnfDGK^M^ via this approach. Finally, there is an obvious reduction in the amount of the *vnfG*-encoded δ subunit in the VnfDGK^M^ protein (Fig. 1A). This observation coincides with results derived from the characterization of a cofactor-deficient form of VnfDGK, which reveals the absence of the δ subunit and an incomplete, αβ_2_-trimeric composition of this cofactor-less protein (27). The positive correlation between the decreased amount of δ subunit and the absence of V-cluster suggests a possible role of the δ subunit in specifically delivering the V-cluster to the cofactor-binding site and maintaining the stability at the α/β subunit interface once its delivery job is finished.

While many aspects related to the expression and assembly of the alternative nitrogenase await investigation, the outcome of this work provides a useful framework for further investigation of the two major determinants—the protein environment and the cofactor species—in order to narrow down the key elements attributing to the CO reactivity of nitrogenase. Moreover, the strategy used in this work for the successful generation of VnfDGK^M^ *in vivo* could potentially be employed for generation of other heterologous forms of nitrogenase, which may facilitate further exploration of this unique reactivity of nitrogenase for potential applications in the future.

## MATERIALS AND METHODS

### Strain construction and cell growth.

*Azotobacter vinelandii* strains YM68A and YM13A (expressing His-tagged VnfDGK and NifDK, respectively) were constructed as described earlier (11, 28). Both strains were grown in 180-liter batches in a 200-liter New Brunswick fermentor (New Brunswick Scientific) in Burke’s minimal medium supplemented with 2 mM ammonium acetate (11, 28). The molybdate in Burke’s medium was replaced by an equal amount of vanadate for the expression of the native VnfDGK^V^ protein in strain YM68A. In preparation for the expression of the VnfDGK^M^ hybrid in strain YM68A, the fermentor was scrubbed with acid and water, followed by growth of two consecutive 180-liter batches of YM68A in Burke’s medium that contained no Mo or V, which permitted removal of trace amounts of V in the vessel. Subsequently, strain YM68A was grown in Burke’s medium supplemented with 2.5-fold molybdate, and cell growth was monitored by measuring the cell density at 436 nm using a Spectronic 20 Genesys spectrophotometer. Cells were harvested in the late exponential phase by a flowthrough centrifugal harvester (Cepa), and the cell paste was washed with a buffer containing 50 mM Tris-HCl (pH 8.0). Published methods were then used for the purification of His-tagged NifDK and VnfDGK and nontagged NifH and VnfH (11, 28).

### Protein characterization and activity assays.

VnfDGK proteins were subjected to sodium dodecyl sulfate-polyacrylamide gel electrophoresis (SDS-PAGE) analysis on a 4 to 20% precast Tris-glycine gel (Bio-Rad). The metal contents of the proteins were determined by inductively coupled plasma-optical emission spectroscopy (ICP-OES) based on previously established protocols (29). All nitrogenase activity assays were carried out as described earlier (30, 31). The hydrocarbon products were analyzed as described elsewhere (4, 5, 29). Ammonium was determined by a high-performance liquid chromatography fluorescence method (32), and hydrogen was analyzed as described previously (33).

### Cofactor extraction and reconstitution of NifDK^apo^.

The NifDK- and VnfDGK^M^-bound M-clusters were extracted into *N*-methylformamide (NMF) using a previously established method (22). The extracted cofactor was then incubated with the M-cluster-deficient, apo-NifDK protein (NifDK^apo^) for 20 min prior to removal of excess metal cluster by passing the reconstituted protein through a G25 column.

### EPR spectroscopy.

EPR samples were prepared in a Vacuum Atmospheres dry box at an oxygen level of <4 ppm. All samples contained 25 mM Tris-HCl (pH 8.0), 10% glycerol, and 2 mM sodium dithionite (Na_2_S_2_O_4_). The EPR spectra were taken in perpendicular mode using a Bruker ESP 300 Ez spectrophotometer (Bruker) interfaced with an Oxford Instruments ESR-9002 liquid helium continuous flow cryostat. All spectra were recorded at 10 K, using a microwave power of 20 mW, a gain of 5 × 10^4^, a modulation frequency of 100 kHz, and a modulation amplitude of 5 G. A microwave frequency of 9.62 GHz was used to collect five scans for each sample.
